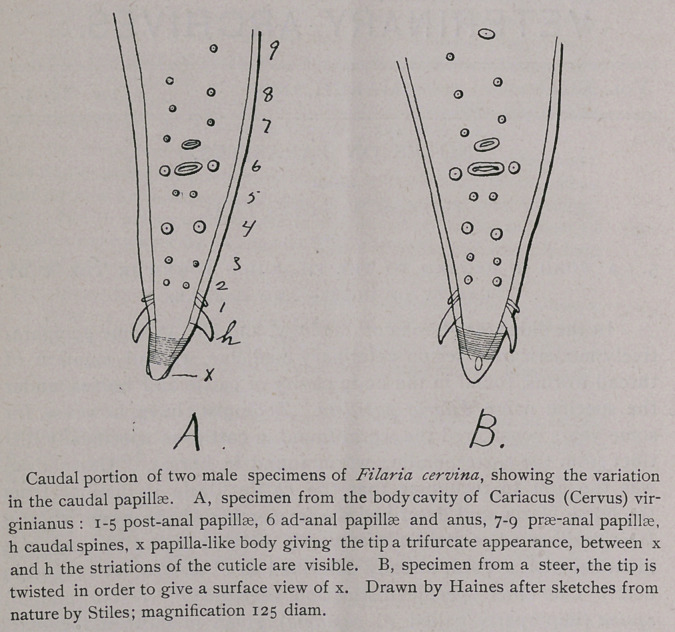# Notes on Parasites

**Published:** 1892-03

**Authors:** Charles W. Stiles


### THE JOURNAL
OF
### COMPARATIVE MEDICINE AND VETERINARY ARCHIVES.

Vol. XIII. MARCH, 1892. No. 3.

### NOTES ON PARASITES.

Charles W. Stiles, Ph.D.

### 5. A WORD IN REGARD TO THE FILARIIDÆ FOUND IN THE BODY CAVITY OF HORSES AND CATTLE.

In the older text-books of Zoölogy, and also in some comparatively modern treatises on veterinary medicine, we find mention of thread worms, found in the body cavity of cattle and horses, under the specific name *Filaria papillosa.* Zoölogists have, however, for some years considered the worm found in cattle as specifically distinct from the corresponding worm found in horses. Alessandrini was the first to recognize that the cattle filaria is distinct from the horse filaria, and described the former under the name *Filaria labio-papillosa ;* but Baillet afterwards noticed that this species is identical with a worm already described as *F. cervina*, and found in deer. The chief specific distinction between *F. cervina* and *F. equina* (improperly called *F. papillosa*) given in most of the text-books, is the presence of four sub-median papillæ on the head of the latter, which are not found on the head of *F. cervina.* Upon studying the species *F. cervina* for the *Report on Animal Parasites of Cattle*, now in preparation in the Bureau of Animal Industry, I noticed a characteristic of both *F. equina* and *F. cervina* which renders the present accepted diagnosis of these two species somewhat incorrect, i. e.: the *presence of four sub-median sense papillæ in both species*, of which I can find no notice in the works at my disposal. Immediately in front of these four sense-papillæ are also four minute spines projecting through the cuticle, which evidently stand in close relation to the sense-papillæ ; this latter statement holds for *F. cervina*, but I have not been able to distinguish them in *F. equina*, although I confidently expect to do so with fresh material. The four so-called papillæ so characteristic of *F. equina* are, as all authors now agree, absent in *F. cervina ;* these are, however, not *sense-papillæ*, but *spine-papillæ*, and in reading the diagnoses and determining these two species of parasites we must remember this distinction.

Another point in which I differ with other authors who have published on *F. cervina* is in regard to the sense-papillæ on the tail of the males. All former authors unite in stating that there are four pairs of præ-anal papillæ. Strictly speaking, this is incorrect, for as my figures show, only three pairs of papillæ are præ-anal, while one pair is at the side of the anus, for which I suggest the designation *ad-anal*. Again, I find *five* pairs of post-anal papillæ instead of four pairs, and one pair of prominent lateral hooks. On the tip of the tail there are also two small lateral projections, so that the tip has a tri-furcate appearance. On comparing my figure with the figure given by Schneider (Monographie der Nematoden, p. 86), it will be seen immediately that it is the fifth pair of papillæ, i. e.: the pair next behind the anus, which has heretofore escaped attention.

The first pair of papillæ are lateral, as Schneider has already described, and are immediately in front of the lateral hooks or spines. My description of the caudal papillæ has been made from a male *F. cervina* taken from a deer, *Cariacus* (*Cervus*) *virginianus*, and has been compared minutely with cattle parasites of the same species, with which it fully agrees. Upon comparing six males, some variation in the relative position of the papillæ was noticed.

At present I cannot make any positive statements in regard to the caudal papillæ of *F. equina*, as the male specimens of that species which were at my disposal were too macerated to warrant a description. It is, however, quite probable that a careful examination will reveal a fifth pair of post-anal papillæ in the males of that species also. As soon as I can obtain proper material, I shall have something more to say both in regard to *F. equina* and *F. cervina*. So far as I can learn, there is no ground for considering that *F. equina* of the horse can occur in cattle, or that *F. cervina* of cattle can occur in horses.

The following is the present view of the synonymy and specific diagnosis of the two parasites :

1. Filaria in the abdominal cavity of cattle. *Filaria cervina* Duj. 1845, (Syn. F. cervi elaphi Rud., F. papillosa Rud. ex parte, F. labio-papillosa Aless., F. terebra Dies. 1851, possibly also F. bubali Rud., and F. tentaculata Mehlis 1846). The names *F. bubali* and *F. cervi elaphi* antedate *F. cervina*, but cannot be accepted, since no recognizable desciptions were given with them. I am unable to obtain the date of publication of Alessandrini's paper, so am obliged to accept the name Dujardin gave to the species ; but as Neumann places Dujardin's name between *F. cervina* Duj. and *F. terebra* Dies., there is no reason for us to doubt that *F. cervina* has the priority.

Description.—Body filiform, whitish, opaque ; anterior portion rather blunt; the base of the mouth frequently appears as a double contoured ring, which does not lie in one plane, but presents four curvatures, the dorsal and ventral curvatures being convex towards the tail, the lateral curvatures concave towards the tail. In both the dorsal and ventral median lines, the chitinous support is raised in twin spine-papiliæ in the female, while in the males the twinning of the spines is not so evident ; on the sides it extends over the mouth in the form of small semi-lunar lips (both sexes). Back of the mouth are four small sub-median round openings in the cuticle, through each of which a *sense*-papilla extrudes, and directly in front of each sense-papilla a minute spine is found. Mouth is dorso-ventrally oblong. Male 4-6 cm. long; three pairs of præ-anal, one pair of ad-anal, and five pairs of post-anal papillæ; tail wound spirally. Female 6-12 cm. long. Tip of tail is blunt and studded with several small knobs. The two lateral spine papillæ are 0.128 mm. form the tip.

It is generally stated that this species differs from the following in the absence of striations of the cuticle, but I do not find this character constant for the entire length of the body, as I have frequently seen undoubted specimens of *F. cervina* with a cuticle which was striated between the tip of the tail and the caudal spines; a pseudo-striation also occurs on the ventral and lateral surfaces of the tail of the males, extending some distance in front of the anus.

2. Filaria in the abdominal cavity of horses. *Filaria equina* (Abildgaard) E. Bl., Syn. according to Diesing, Gordius equinus Abildgaard, Filaria equi Gmelin, *F. papillosa* Rud. (ex parte, Aless), *Ascaris pellucida* Brown, Thelazia Rhodesii Desmarest, 1828, F. equina E. Blanchard.)

As will be seen from the synonymy given above, this parasite was first described under the name Gordius equina; Gmelin and Rudolphi discovered that it belonged to the genus Filaria instead of Gordius, and named it *Filaria equi* and *Filaria papillosa* respectively. This latter name has been accepted by nearly every author who has mentioned the worm since the time of Rudolphi, but E. Blanchard and Railliet revert to the specific name *equina*, and since that has the priority, there can be no doubt but that we should follow it in this case.

Description.—Scarcely distinguishable from the former species except with the aid of a magnifying glass or a microscope. Body white, attenuated towards the extremities. Mouth small, supported by a chitinous border which generally shows the double contoured ridge, where it is joined to the body, less prominently than is the case in *F. cervina;* it gives rise to two semi-lunar lips which border the mouth laterally; in both the dorsal and ventral median lines it projects in a spine-papilla, which is not twinned as in *F. cervina*, although in some specimens I discovered a slight indentation on the apex, which if carried further would have resulted in twin papillæ. The anterior portion is very blunt, near the border of the blunt portion are found four sub-median strong chitinous spine-papillæ, and this forms one of its most distinguishing characters. Back of the blunt anterior extremity are found four sub-median sense-papillæ, such as we described in *F. cervina.* The *male* is 6-7 cm. long, and its spirally curved tail possesses four pairs of præ-anal and four (five?) pairs of post-anal papillæ, of which No. 1 is conical and turned towards the side. The two spicules are unequal. The *female* is 9-12 cm. long, the tail slightly spiral and terminated with a much shorter conical projection than is the case in *F. cervina*. The lateral spines are 0.064 mm. from the tip. Vulva very near the mouth, uterus double. Ovoviviparous. *F. equina* is found in the horse, ass and mule. As already stated, some authors report it also from cattle; but we are inclined to believe that they have failed to distinguish this form from *F. cervina*. *F. equina*, like *F. cervina*, is found in the peritoneal cavity, more rarely in the skull. It is also said to wander in the substance of the brain and spinal cord.

A fuller discussion of these parasites will be published in the Bureau reports.

*Bureau of Anmial Industry, U. S. Department of Agriculture, Jan. 7, 1892.*

6. ON THE PRESENCE OF STRONGYLUS OSTERTAGI (OSTERTAG, 1890) STILES 1892, in America.

In two publications of 1890, Prof. Ostertag described a new species of Strongyle (7-13 mm. long) from the fourth stomach of cattle, under the name *Str. convolutus.* The only other publication known to me in regard to this worm is an anatomical description by Dr. Stadelmann, of the Berlin Zoölogical Institute, and as all three of these papers are based upon parasites found in Berlin, we know very little as yet in regard to the geographical distribution of this helminth. In America we find a worm causing small ulcers in the fourth stomach of cattle and sheep, which, although the measurements do not agree in all particulars with those given by the German authors, I have no hesitation in diagnosing as identical with Ostertag's *Str. convolutus.* We must, however, take exception to the name which is employed in Europe to designate this worm, since the specific name *convolutus* has long been pre-occupied in the genus *Strongylus*, having been used by Kuhn (Memoir. d. mus. d'hist. nat. XVII.), in connection with a parasite found in the bronchial tubes of *Phocæna communis* (Delphinus phocæna). Kuhn's species has since been placed in another genus as *Pseudalus convolutus*, but the name *Str. convolutus* exists in older books, and is now used as a synonym. It is on this account that I have thought best to introduce a new specific name for Ostertag's species, naming it after its discoverer. The parasite appears to be rather common, in some parts of this country at least, for I found it in the recent Blairsville epizootic among sheep, and Drs. Smith, Hassall and Curtice, as well as myself, have found it in Washington both in animals at the Bureau Experiment Station and at the slaughter house.

Ostertag found this worm in 90 per cent. of the cattle slaughtered in Berlin, Prussia.

Slight infections produce no appreciable effects upon the host, but heavy infections produce a more or less extensive catarrhal affection of the stomach. Forms of the parasite in all stages of development are found in the small ulcers of the epithelium, the ulcers ranging from the size of a pin-head to that of a small pea, while the older worms are frequently seen free on the epithelium, and are somewhat difficult to distinguish from young specimens of *Str. contortus*, (the twisted strongyle). The two worms are nearly allied, but a microscopical examination shows them to be distinct species, for while the bursa in the male of *Str. contortus* is distinctly bilobed, with a small asymmetrical dorsal lobe, the bursa in *Str. Ostertagi* is continuous. The spicules of the males in the two species are also very different. A more detailed accoun tof these parasites will appear in the reports of the Bureau of Animal Industry.

*U. S. Department of Agriculture, Jan. 20, 1892.*

7. A WORD IN REGARD TO DR. FRANCIS'S *Distomum texanicum.*

No. 7 of this series of short papers on parasites appears this month in the *American Veterinary Review.* In it I state that Dr. Francis's new species of liver-fluke found in Texas cattle (*D. texanicum* Francis, October, 1891,) is identical with *D.* (*Fasciola*) *americanum* Hassall (September, 1891), hence the name given to the parasite by Dr. Francis must be dropped as a specific name and be regarded as a synonym. It is also stated in the note that there is no doubt in my own mind that *D. americanum* Hassall is identical with *D. magnum*, a large fluke which was described some years ago by Prof. Bassi, and which since that time has generally been considered as identical with *D. hepaticum.* To Dr. Hassall is due the credit of insisting upon a specific distinction between *D. hepaticum* and the large fluke found in American cattle, and this is not lessened in the slightest because I wish to make this form identical with *D. magnum* Bassi, a view, I may add here, which is also supported by Leuckart and Blanchard (personal correspondence). The synonymy given in the note is: *Distomum magnum* Bassi 1875, as specific name, (syn.) *Fasciola carnosa* Hassall (July, '91), *F. americana* Hassall (Sept., 1891) and *D. texicanum* Francis (Oct., 1891). Should it be proven that *F. americanum* is not identical with *D. magnum*, then Dr. Hassall's specific name must stand in preference to the one given by Dr. Francis, the form being *Distomum americanum.*

*U. S. Dept. of Agriculture, Jan. 30, 1892.*

## Figures and Tables

**Figure f1:**